# Central Hypoventilation: A Case Study of Issues Associated with Travel Medicine and Respiratory Infection

**DOI:** 10.1155/2015/647139

**Published:** 2015-07-29

**Authors:** Kam Lun Hon, Alexander K. C. Leung, Albert M. C. Li, Daniel K. K. Ng

**Affiliations:** ^1^Department of Paediatrics, The Chinese University of Hong Kong, Prince of Wales Hospital, Shatin, Hong Kong; ^2^Department of Pediatrics, University of Calgary, Calgary, AB, Canada T2M 0H5; ^3^Department of Paediatrics, Kwong Wah Hospital, Kowloon, Hong Kong

## Abstract

*Aim*. We presented the case of a child with central hypoventilation syndrome (CHS) to highlight issues that need to be considered in planning long-haul flight and problems that may arise during the flight. *Case*. The pediatric intensive care unit (PICU) received a child with central hypoventilation syndrome (Ondine's curse) on nocturnal ventilatory support who travelled to Hong Kong on a make-a-wish journey. He was diagnosed with central hypoventilation and had been well managed in Canada. During a long-haul aviation travel, he developed respiratory symptoms and desaturations. The child arrived in Hong Kong and his respiratory symptoms persisted. He was taken to a PICU for management. The child remained well and investigations revealed no pathogen to account for his respiratory infection. He went on with his make-a-wish journey. *Conclusions*. Various issues of travel medicine such as equipment, airline arrangement, in-flight ventilatory support, travel insurance, and respiratory infection are explored and discussed. This case illustrates that long-haul air travel is possible for children with respiratory compromise if anticipatory preparation is timely arranged.

## 1. Introduction

Travel medicine is the branch of medicine that deals with the prevention and management of health problems of international travelers [1–4]. We presented the case of a child with central hypoventilation syndrome (CHS) to highlight issues that need to be considered in planning long-haul flight and problems that may arise during the flight.

## 2. Case

In the summer of 2013, the pediatric intensive care unit (PICU) of a hospital in Hong Kong received an 8-year-old boy with central hypoventilation with respiratory infection and decompensation en route to Hong Kong on a make-a-wish campaign. He was diagnosed with central hypoventilation (medullary atrophy) or Ondine's curse and had been well managed in Toronto, Canada. He was ambulatory, only needed home ventilatory support at night via tracheostomy, and inhaled salbutamol puffs on a* prn* basis, and he was on PEG (percutaneous endoscopic gastrostomy) feeding with puree food. Advanced fitness for air-travel arrangement was well negotiated with the respective commercial airline. However, he developed symptoms of respiratory infections with intermittent fever (up to 39°C), cough, and sputum for 2 days prior to departure. The child was seen at the emergency department of a children's hospital in Toronto and was treated with an oral course of cefuroxime. During the long-haul flight, symptoms of respiratory infections persisted and desaturations (86%) developed. The patient had his own oxygen monitoring and air compressor on board which needed to be increased to 1 L/min. On arrival in Hong Kong, he was taken to the emergency department. His vital signs were as follows: tympanic temperature 38.6°C, heart rate 157/min, and SpO_2_ 98% on own ventilator with flow 1 L/min. The home ventilator's electric plug was in Canadian style and did not fit the Hong Kong standard socket. Chest radiograph revealed mild right sided haziness. He was admitted to PICU for management. He weighed 23.2 kg and his vital signs were as follows: temperature 36.5°C, heart rate 121/min, respiratory rate 23/min, BP 97/57 mmHg, and SpO_2_ 97% in room air on arrival at PICU. The child received physiotherapy and the tracheostomy was temporarily connected to the ICU ventilator on SIMV mode with pressure control (PC) and pressure support (PS). Settings were FiO_2_ 0.25, inspiratory time (Ti) 0.9 seconds, intermittent mandatory ventilation (IMV) rate 20/min, positive end expiratory pressure (PEEP) 5 cm H_2_O, pressure control 15 cm H_2_O above PEEP, and PS 13 cm H_2_O above PEEP. There was no further desaturation, and the settings were gradually reduced to IMV 10/min and FiO_2_ of 0.21. The child gave a history of drug allergy to Ativan (lorazepam) and gluten sensitivity. Sedation was not needed. He received a course of intravenous amoxicillin/clavulanate (30 mg/kg/dose, 8 hourly). The patient remained playful, talkative, and not in distress. Laboratory data were normal complete blood count with white blood cell count of 16.1 × 10^9^/L, neutrophil differential of 77%, and elevated C-reactive protein of 44.1 (normal < 9.9 mg/L). There was no bacterial or respiratory viral isolation in the tracheal aspirate. Blood culture was negative. The patient was discharged from the PICU 2 days later and went on with his make-a-wish journey to Disneyland in Hong Kong prior to returning home.

## 3. Discussion

Ondine's curse, also called congenital central hypoventilation syndrome (CCHS) or primary alveolar hypoventilation, is a serious form of central nervous system failure, involving an inborn failure of autonomic control of breathing. Patients generally require tracheotomies and lifetime mechanical ventilator support. With advances of home ventilatory support, patients with central hypoventilation are no longer “cursed.” They can live a relative normal life at home, as reported by Hon et al., even in the remote countryside setting in one case report [5–7].

Travel medicine is the branch of medicine that deals with the prevention and management of health problems of international travelers [1–4]. The field of travel medicine encompasses a wide variety of disciplines including epidemiology, infectious disease, public health, tropical medicine, high altitude physiology, travel related obstetrics, psychiatry, occupational medicine, military and migration medicine, and environmental health. In our case, potential problems that may arise during travel include cardiopulmonary disease mortality, injury, and accident. Infectious disease accounts for about 2.8–4% of deaths during/from travel [1–4, 8–10]. In terms of morbidity, traveler's diarrhea is the most common problem encountered [9, 10].

In this day and age, international travel is made possible even for patients who need ventilatory support. Prior to a long-haul air travel, parents should negotiate with airline to detail the transport and inflight plans [8, 11, 12]. The following part gives 4 website addresses of checklists for commercial air-travel preparation for ventilated children. In our case of CCHS with specific needs for ventilatory support, the patient, family, and the airline collaborated well. In general, the patient's usual emergency medication such as inhaled salbutamol should be readily accompanying the patient. Additional space is required to station the ventilator, air compressor, and monitor. An international travel insurance policy is mandatory in this age of unexpected and unavoidable disasters [8].

Websites of checklists for commercial air-travel preparation for ventilated children are as follows:

1. A Special Needs Preflight Checklist: 16 Things You Need to Do before Heading to the Airport (http://www.friendshipcircle.org/blog/2012/01/09/a-special-needs-pre-flight-checklist/).
  - Booking your tickets:
    - Stopover or direct flight?
    - Best time of day to travel.
    - Choose airline wisely.
    - What seat is best for your child?
  - Medical preparation:
    - Travel prescriptions.
    - A letter from a doctor.
    - Medications and medical records.
    - Medical equipment.
    - In case of emergency.
  - Preparing your child to fly:
    - Read about airports and airplanes.
    - Airplane videos.
    - Social stories.
    - Airport visits.
    - Mock flights.
  - Before you head to the airport:
    - Call Transportation Security Administration (TSA) in the USA.
    - Small bills.
    - Check in at home.
    - Have a backup plan.
    - Take a deep breath and smile.
2. Medical Guidelines for Airline Passengers, Aerospace Medical Association, Alexandra, VA (May, 2002) (http://www.asma.org/asma/media/asma/Travel-Publications/paxguidelines.pdf).
  - General advice:
    - Have all medication in carry-on luggage and be sure it is in its original container with the prescription label.
    - If you have significant medical problems, carry an abbreviated copy of your medical records.
    - Alert airlines in advance of special requirements.
    - Wear loose comfortable clothing.
    - Allow extra time.
    - Consider buying insurance which includes provision for air evacuation home in event of any medical condition.
3. Travel with a Ventilator: Ventilation Resource Site (http://www.livingwithavent.com/pages.aspx?view=noninv&page=Living/Travel).
  - Talk with other ventilator users.
  - Consider power.
  - Include supplies.
  - Check transportation procedures:
    - Plan travel well in advance.
    - Obtain approval for in-flight ventilation.
    - Get to the airport with ample time before departure.
    - Protect the ventilator.
    - Bring adequate power for the use of the ventilator in flight, or check if the aircraft has outlets for medical use.
    - Check oxygen availability during flight.
    - Prepare for possible technical problems with the ventilator.
  - Know ventilator settings.
4. Air Travel and Ventilator Users, International Ventilator Users Network, 2003 (http://www.ventusers.org/edu/valnews/val17-3c.html#air).
   This website gives some tips about travelling with a ventilator.

One more issue pertinent to the discussion of travel medicine is advice regarding feasibility of long-haul travel in a patient with acute or intercurrent respiratory infection. Since the days of SARS (severe acute respiratory syndrome) and recently MERS (Middle East respiratory syndrome) and SARI (severe acute respiratory infection), travel transmission of novel respiratory infections has become hot issues [13–15]. Ideally, patients with an acute respiratory infection should not be travelling for the patient's own sake as well as for the sake of other passengers. However, travel may be once-in-a-life-time opportunity for a young person with chronic illness as was in our case. This may be inhumane to the child if his/her opportunity was removed from him/her due to a non-life threatening chest infection. On the other hand, the risk has to be weighed between a seemingly minor infection which may predispose a major decompensation during long-haul travel in a child with already compromised respiratory health.

No published literature on mortality and morbidity in children travelling with central hypoventilation syndrome or Ondine's curse is available. Critically ill children are transported safely via medical evacuation teams [16, 17]. However, critically ill children cannot be transported via commercial airlines. Our case illustrates that children who are ventilator dependent may travel in commercial airlines if they are stable. The issue of quarantine to prevent international transmission of SARI is a much more complicated issue that is likely to remain a contemporary controversy. This case illustrates the many issues associated with long-haul flight in a pediatric patient with a chronic respiratory disorder. Air travel is possible for children with respiratory compromise if anticipatory preparation is timely arranged.

## References

[1] Cupa M. (2009). Air transport, aeronautic médecine, health. *Bulletin de l'Academie Nationale de Medecine*.

[2] Al-Zurba F., Saab B., Musharrafieh U. (2007). Medical problems encountered among travelers in Bahrain International Airport clinic. *Journal of Travel Medicine*.

[3] Eray O., Kartal M., Sikka N., Goksu E., Yigit O. E., Gungor F. (2008). Characteristics of tourist patients in an emergency department in a Mediterranean destination. *European Journal of Emergency Medicine*.

[4] Felkai P. (2008). Analysis of prevention in travellers diseases on the basis of latest results in travel medicine. *Orvosi Hetilap*.

[5] Hon E. K., Wilson M., Hindle R. C. (1994). The survival story of a child with Ondine's curse in Northland. *New Zealand Medical Journal*.

[6] Juan G., Ramon M., Ciscar M. A. (1999). Acute respiratory insufficiency as initial manifestation of brain stem lesions. *Archivos de Bronconeumología*.

[7] Wang T.-C., Su Y.-N., Lai M.-C. (2014). PHOX2B mutation in a Taiwanese newborn with congenital central hypoventilation syndrome. *Pediatrics and Neonatology*.

[8] Cocks R., Liew M. (2007). Commercial aviation in-flight emergencies and the physician. *Emergency Medicine Australasia*.

[9] Harvey K., Esposito D. H., Han P. (2013). Surveillance for travel-related disease—GeoSentinel Surveillance System, United States, 1997–2011. *Morbidity and Mortality Weekly Report. Surveillance Summaries*.

[10] Kollaritsch H., Paulke-Korinek M., Wiedermann U. (2012). Traveler's diarrhea. *Infectious Disease Clinics of North America*.

[11] Matthys H. (2011). Fit for high altitude: are hypoxic challenge tests useful?. *Multidisciplinary Respiratory Medicine*.

[12] Basu A. (2007). Middle ear pain and trauma during air travel. *BMJ Clinical Evidence*.

[13] Hon K. L. (2009). Just like SARS. *Pediatric Pulmonology*.

[14] Hon K. L. (2013). Severe respiratory syndromes: travel history matters. *Travel Medicine and Infectious Disease*.

[15] Lim P. L., Lee T. H., Rowe E. K. (2013). Middle east respiratory syndrome coronavirus (MERS CoV): update 2013. *Current Infectious Disease Reports*.

[16] Hon K. L., Olsen H., Totapally B., Leung T.-F. (2005). Hyperventilation at referring hospitals is common before transport in intubated children with neurological diseases. *Pediatric Emergency Care*.

[17] Hon K.-L. E., Olsen H., Totapally B., Leung T.-F. (2006). Air versus ground transportation of artificially ventilated neonates: comparative differences in selected cardiopulmonary parameters. *Pediatric Emergency Care*.

